# Advances of Engineered Hydrogel Organoids within the Stem Cell Field: A Systematic Review

**DOI:** 10.3390/gels8060379

**Published:** 2022-06-15

**Authors:** Zheng Li, Muxin Yue, Yunsong Liu, Ping Zhang, Jia Qing, Hao Liu, Yongsheng Zhou

**Affiliations:** 1 Department of Prosthodontics, Peking University School and Hospital of Stomatology & National Center of Stomatology & National Clinical Research Center for Oral Diseases & National Engineering Research Center of Oral Biomaterials and Digital Medical Devices & Beijing Key Laboratory of Digital Stomatology, 22 Zhongguancun South Avenue, Haidian District, Beijing 100081, China; pkulizheng@126.com (Z.L.); yuemuxinpku@163.com (M.Y.); liuyunsong@hsc.pku.edu.cn (Y.L.); zhangping332@bjmu.edu.cn (P.Z.); qingjia0723@163.com (J.Q.); 2 The Central Laboratory, Peking University School and Hospital of Stomatology & National Center of Stomatology & National Clinical Research Center for Oral Diseases & National Engineering Research Center of Oral Biomaterials and Digital Medical Devices & Beijing Key Laboratory of Digital Stomatology, 22 Zhongguancun South Avenue, Haidian District, Beijing 100081, China

**Keywords:** organoid, hydrogel, stem cells, tissue engineering

## Abstract

Organoids are novel in vitro cell culture models that enable stem cells (including pluripotent stem cells and adult stem cells) to grow and undergo self-organization within a three-dimensional microenvironment during the process of differentiation into target tissues. Such miniature structures not only recapitulate the histological and genetic characteristics of organs in vivo, but also form tissues with the capacity for self-renewal and further differentiation. Recent advances in biomaterial technology, particularly hydrogels, have provided opportunities to improve organoid cultures; by closely integrating the mechanical and chemical properties of the extracellular matrix microenvironment, with novel synthetic materials and stem cell biology. This systematic review critically examines recent advances in various strategies and techniques utilized for stem-cell-derived organoid culture, with particular emphasis on the application potential of hydrogel technology in organoid culture. We hope this will give a better understanding of organoid cultures for modelling diseases and tissue engineering applications.

## 1. Introduction

Due to their extensive capacity for self-renewal and multipotent differentiation, stem cells are widely utilized in regenerative medicine and developmental biology research, in addition to modeling the pathophysiological progression of diseases. Recent advances in 3D cell culture techniques enable the development of in vitro tissue models, also known as “organoids”. Micromachining, 3D printing, and hydrogels are the basic elements of 3D cell culture techniques [1]. Various 3D culture methods for different tissues have been developed in recent years, which laid a solid foundation for the development of organoid culture techniques [2,3,4,5,6,7,8,9]. Stem-cell-derived organoids are in vitro organ models that form functional tissue types by self-organization and differentiation, which can potentially facilitate a better understanding of the biology and physiology of various organs [10]. Engineered organoids could help build biological sample biobanks, construct disease models to study disease pathogenesis, be utilized for therapeutic drug screening, and could also be applied in regenerative medicine and organ repair [11]. For example, organoids could model the pathology of infectious diseases such as COVID-19, which could contribute to the development of better therapeutic modalities [12].

To date, a number of studies have reported that stem-cell-derived organoids using hydrogels could simulate many different types of tissues and organs, including the intestine [13,14,15], brain [16,17,18], retina [19,20], cardiac tissues [21,22,23], kidney [24,25,26], lung [27,28], various oral tissues with (tooth germ organoids [29], lingual epithelium organoids [30], salivary gland [31] and taste bud organoids [32]), liver [33,34], cartilage [35], and vascular tissues [17]. The most commonly used hydrogel is Matrigel, which plays a key role in 3D culture organoid technology due to it being enriched in various growth factors and extracellular matrix components (such as laminin, type 4 collagen, growth factors, etc.). However, due to its tumor-source origin, Matrigel faces challenges in being approved by the FDA, which greatly limits the clinical translation and application of organoids in the biomedical field [36]. In addition, Matrigel differs greatly from the extracellular matrix (ECM) environment of normal tissues and organs and cannot provide a tissue-specific ECM for stem cells. Due to these limitations, more research has attempted to find alternatives to Matrigel [36].

Organoids could be derived from somatic cells, Pluripotent stem cells (PSCs), or adult stem/progenitor cells from specific organs. PSCs-derived organoids could be established after PSCs-oriented differentiation that requires induction and maturation of the endoderm, mesoderm, or ectoderm, followed by culture with specific growth and signalling cytokines to obtain the desired tissue type. Adult stem-cell-derived organoids require tissue-specific stem cells to be isolated and then embedded within the extracellular matrix and require a specific sequence of growth factors or cytokines to regulate differentiation [37]. State-of-the-art CRISPR tools enable genome editing of human stem cells. Using this technology, gene-edited or mutated PSCs or somatic cells with altered signaling pathways provide novel insights into the molecular mechanisms of tissue development and homeostasis, in addition to enabling precise control of organoid development [38].

In summary, organoids have emerged in recent years as an outstanding in vitro model, not only for disease modelling but also for tissue engineering, drug toxicology, clinical immunity, and other fields [12]. One ultimate goal of organoid technology is to construct a bio-smart organoid, which can be well-integrated with native tissues to repair damaged organs. In order to better explore the potential application of organoids, scientists should make further efforts in the development of advanced bio-smart materials together with a deeper understanding of stem cell biology [39].

Therefore, this work systematically reviews recent advancements in stem-cell-derived organoid engineering strategies based on hydrogels. The application of organoids derived from adult stem cells (ASCs) and PSCs will be examined with the aim of helping scientists efficiently choose appropriate stem cell sources. Then the divergence of construction strategies with regard to different organs will be discussed. Furthermore, current challenges and perspectives of stem-cell-based organoids, including their limitations and further directions for clinical applications, will be examined. A systematic understanding of generating organoids from stem cells and hydrogels would help to accelerate their biomedical applications.

## 2. Methods

### 2.1. PRISMA Statement

**Figure 1:**
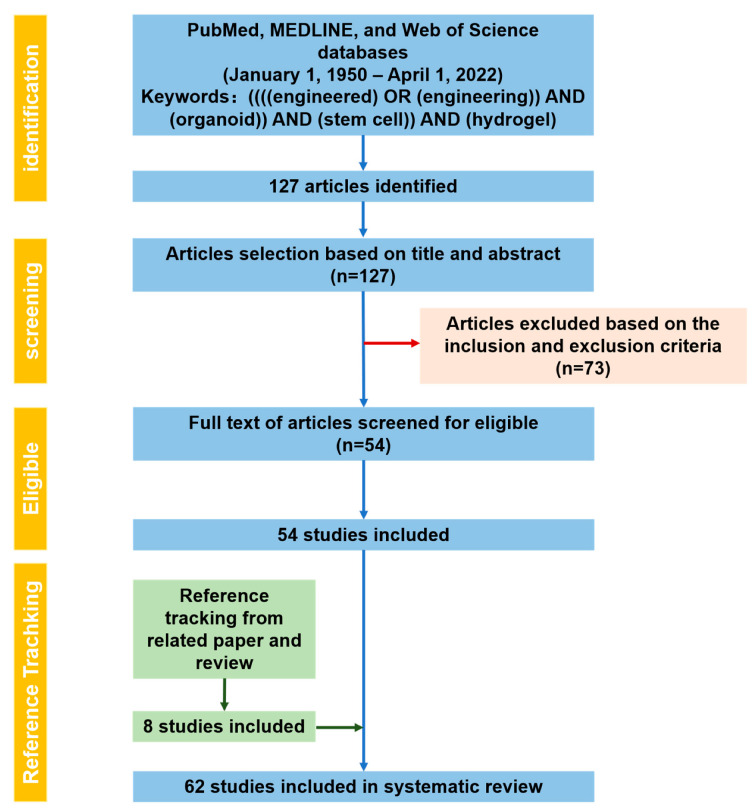
PRISMA flowchart for study selection.

We have finished the PRISMA 2020 checklist and constructed a flowchart, following the PRISMA guidelines and registration information (the registration ID is 338444). The selection process was based on the PRISMA statement 2020 [40], and the flowchart is shown in Figure 1.

### 2.2. Research Process

All relevant papers written in English were searched within PubMed, MEDLINE, and the Web of Science (1 January 1950–1 April 2022). The keywords and their combination were as follows: ((((engineered) OR (engineering)) AND (organoid)) AND (stem cell)) AND (hydrogel). The abstracts of the studies were screened based on the following inclusion and exclusion criteria.

Inclusion criteria: (1) Only original research articles were included. (2) Studies focused on the application of stem-cell-derived organoids in tissue engineering and regenerative medicine, disease modelling, genome editing, and omics analysis (transcriptomics, proteomics, epigenomics, and metabolomics) of healthy and diseased organoids were included.

Exclusion criteria: (1) duplicate studies; (2) books, reviews, congress and conference abstracts, systematic reviews, meetings, short communications, letters, literature updates, and laboratory protocols or articles that only focused on techniques; (3) studies that investigated the application of stem-cell-derived organoids in host–microbe interactions, drug screening, and toxicity testing, personalized therapy and biobanking of patient-derived organoids; tumor therapy; (4) studies not using stem cells or hydrogels; (5) articles whereby full text is not available (abstract only).

All the abstracts of selected articles were evaluated as the inclusion and exclusion criteria described by two researchers, respectively. If the abstract and title were not related to the topic or did not allow the inclusion criteria, the article was excluded. This led to the search of 54 articles fulfilling the eligibility criteria. For further screening, the full texts of the remaining articles were read. When all the related articles were screened, the reference tracking process was completed. A total of 62 papers were included in this systematic review.

### 2.3. Risk of Bias

Once the initial query search was started, the risk of bias existed because the search scopes only included publication dates between 1 January 1950 to 1 April 2022. Moreover, bias existed during the search process because the inclusion and exclusion criteria were restricted by the authors themselves. In addition, the database was defined as including only PubMed, MEDLINE, and the Web of Science, so there could be publications in other databases which would be missed in the selection process. In addition to the aforementioned biases, this systematic review has focused only on scientific publications involving stem-cell-derived organoids using hydrogels.

## 3. Results

### Literature Search Output

Based on the included studies, there are three key elements in the construction of stem-cell-derived hydrogel organoids: stem cells, hydrogels, and construction techniques. In general, when implanted in a 3D microenvironment, stem cells exhibit an intrinsic ability to form complex structures. Although it is not necessary to provide a scaffold (such as hydrogels) to generate organoids, 3D ECM still plays a critical role in building a majority of organoids. In the organoid formation process, appropriate stem cell sources are another key factor, and the mechanical and physical properties of the stem cell microenvironment could regulate stem cell behaviors.

Among the 62 included studies (Figure 1), the most common types of stem cells utilized were iPSCs (12 studies), embryonic stem cells (ESCs) (9 studies), PSCs (11 studies), ASCs (21 studies), other types of stem cells or co-cultures of different cell types (6 studies) and combination of iPSCs, PSCs and/or ESCs (3 studies).

**Figure 2:**
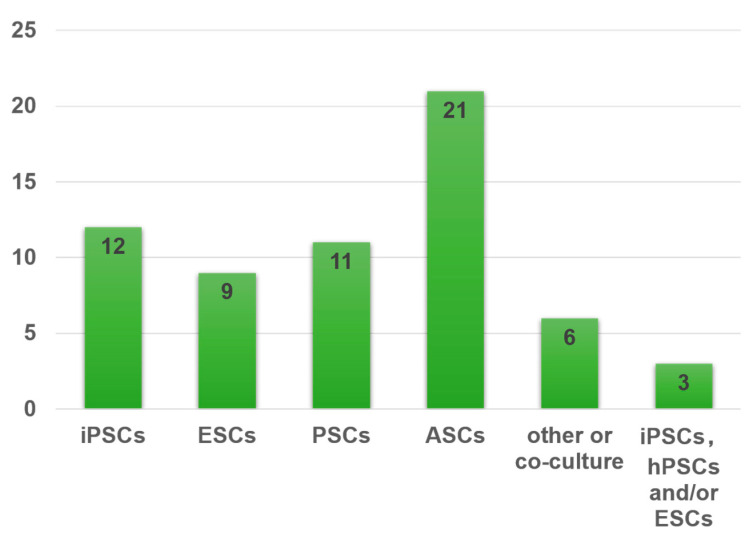
A histogram revealing the number of included articles using different stem cells. Abbreviations: ASCs: adult stem cells; iPSCs: induced pluripotent stem cells; ESCs: embryonic stem cells; PSCs: pluripotent stem cells.

Among the included articles, 35 studies utilized natural hydrogels, 19 studies used synthetic hydrogels, while 8 studies used hybrid hydrogels. Amongst these, the most frequently used natural hydrogels were Matrigel, while alginate, collagen, cellulose, hyaluronic acid (HA), fibrin, gelatin, and decellularized extracellular matrix (dECM), and elastin-like protein hydrogels were also used. The most frequently used synthetic polymer was PEG and its derivatives, gelatin methacrylate (GelMA), while polyacrylamide (PAAm), amikagel were also listed. Among hybrid hydrogels, which included natural-natural hydrogels, synthetic-synthetic hydrogels, and natural-synthetic hydrogels, there were three studies, one study, and four studies, respectively (Figure 2).

**Figure 3:**
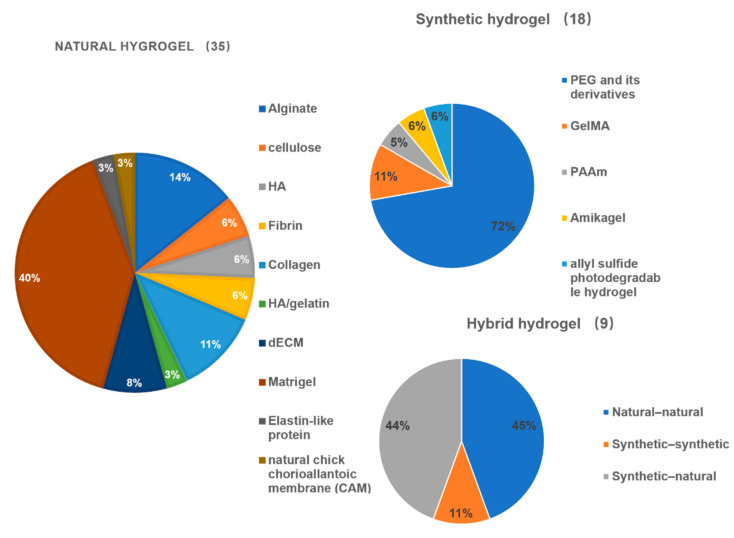
A pie chart showing the number and proportion of included articles using natural hydrogels, synthetic hydrogels, and hybrid hydrogels. Abbreviations: HA: hyaluronic acid; CAM: chorioallantoic membrane; dECM: decellularized extracellular matrix; PEG: polyethylene glycol; GelMA: gelatin-methacryloyl; PAAm: polyacrylamide gels.

**Figure 4:**
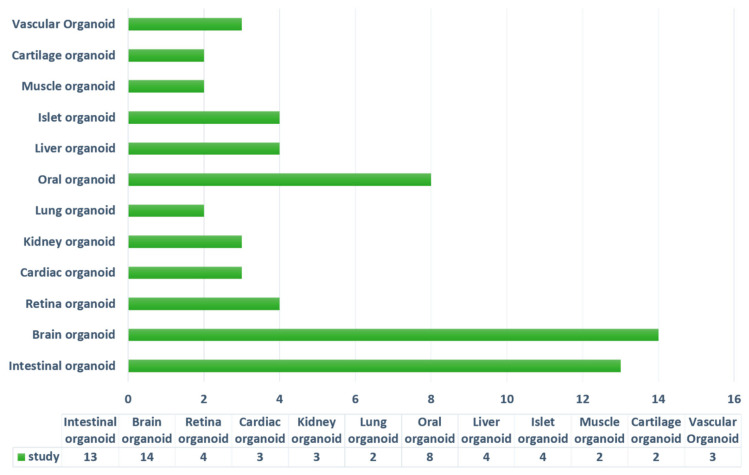
A bar chart representing the number of different types of organoids within the included articles.

**Figure 5:**
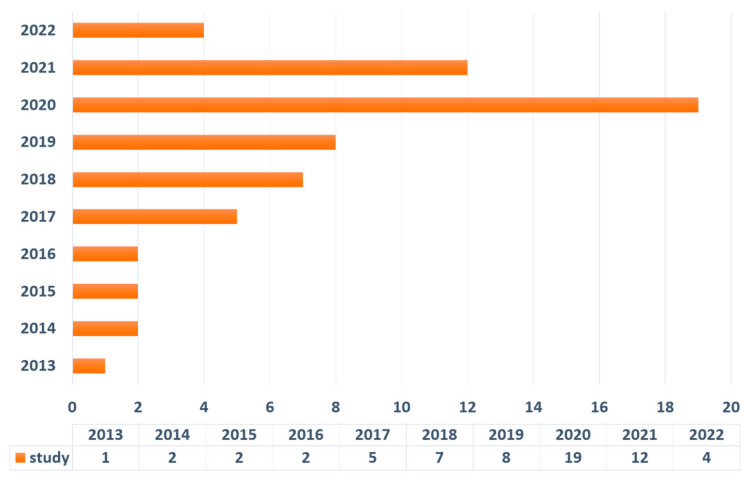
Publication years of included studies.

Among the 62 studies that were included (Figure 3), 12 types of tissue organoids were reported. As illustrated in Figure 4, 13 studies focused on intestinal organoids, 14 studies constructed brain or neural tube organoids, 4 studies investigated retina organoids, 3 studies discussed cardiac organoids, 3 studies focused on kidney organoids, 2 studies explored lung organoids, 8 studies investigated oral organoids (1 study for tooth germ organoids, 3 studies for salivary gland, 1 study for lingual epithelium organoids, and 3 studies for taste bud organoids), 4 studies constructed liver organoids, 4 studies explored liver organoids, 2 studies investigated muscle organoids, 2 studies investigated cartilage and vascular organoids, respectively. All the specific information of the included studies is listed in Table 1. Figure 5 outlines the publication years of related studies.

## 4. Discussion

According to the publication years of the included papers summarized in Figure 5, it is evident that stem-cell-derived hydrogel organoids, especially in the regenerative medicine field, have not been extensively investigated. Therefore, more research in this field should be conducted to enable the advancement of organoid engineering techniques. In this systematic review, the construction techniques relating to different types of tissues based on stem cells and hydrogels are summarized and discussed below.

### 4.1. Representative Types of Stem Cells for Organoids

Stem cells are the main cell sources that are important for tissue development, renewal, and other essential life activities. The behavior of stem cells could be orchestrated by the local stem cell microenvironment around them, called the “niche” [80]. Generally, stem cells that are used for organoid construction can be classified into two major types: (i) pluripotent stem cells (PSCs), including induced pluripotent stem cells (iPSCs) and embryonic stem cells (ESCs), and (ii) adult stem cells (ASCs). In this discussion section, ESCs and iPSCs are collectively termed pluripotent stem cells (PSCs). The construction strategy of organoids is based on the multipotent differentiation and the self-renewal capacity of stem cells. With a deeper understanding of ECM, 3D culture technology can be an effective tool for the structural support of organoids, and the construction methods are diverse and may be customized [39]. Recently, a range of growth factors and small molecules which simulate the microenvironment of tissue-related stem cells during the process of organogenesis have been revealed. ASCs, PSCs, and iPSCs all show a potent tendency to differentiate when exposed to the appropriate ecological niches [81].

#### 4.1.1. Pluripotent Stem Cells (PSCs)

Despite the various limitations of the cell differentiation process, PSCs possess an unlimited self-renewal capacity that allows them to be easily expanded and become an unlimited source of cells for clinical applications [82]. Although the production of iPSCs is expensive and time-consuming, its utilization overcomes the ethical problems related to the clinical use of embryonic stem cells and offers the possibility of using autologous immunocompatible cells for tissue engineering [83].

Organogenesis mimics the natural developmental process from embryo to mature tissue, and it is crucial that appropriate cues exist at the genetic level for organogenesis. Researchers commonly use gain-and-loss-of-function methods to identify which gene is essential for specific lineage function during developmental process. The rapid progression of genome editing techniques of PSCs (ESCs and iPSCs), most notably the CRISPR-Cas9 system, offers a novel and versatile tool for manipulating and exploring organoid modelling [38]. To build organoids, isogenic variants were genetically modified via the novel CRISPR/Cas9 systems to construct specific mutant stem cell types. Moreover, lentivirus vectors or small interfering RNAs (siRNAs) targeting specific genes could be transfected into stem cells to modify genes. One study constructed a PSCs-derived kidney organoid with this model using α-galactosidase. A (GLA)-knock-out hPSCs generated from CRISPR/Cas9, was used to promote the recruitment of endothelial cells to the mouse kidney and enhance interlacement of the vascular network [25]. Nair et al. generated forebrain organoids and explored the effects of human endogenous retroviruses HERV-K(HML-2) using the H9-CRISPRa-HERVK(HML-2) system. They found that the high expression of HERV-K(HML-2) results in hyperactivation of neurodegeneration-related genes, thus exerting an important physiological effect on cortical layer formation in forebrain organoids [16]. A study focusing on retinal organoids using CRISPR/Cas9-derived RB1-null human embryonic stem cells (hESCs) revealed that the cell cycle regulator Retinoblastoma 1 (RB1) played an essential role in early human retina development. The results showed that depletion of RB1 leads to apoptosis and induces cells to enter the S-phase [20]. Generally speaking, these studies suggest that the genome-editing technique is a useful tool for the exploration of PSCs-derived organoids, especially in the construction of disease models and tissue engineering. Additionally, further utilization of genomic technologies such as the CRISPR/Cas9 system could be beneficial for the understanding of specific molecular signaling pathways involved in the formation of organoids derived from PSCs.

#### 4.1.2. Adult Stem Cells (ASCs) or Progenitor Cells

The formation process of PSCs-derived organoids is complicated because it occurs only during embryonic development. Generally, the organogenesis from PSCs takes a relatively long time, for example, about 2–3 months [84]. By contrast, due to the committed lineage of ASCs, the process of differentiation in the organoids usually takes a relatively short time, e.g., at most 3–5 days. Compared with PSCs-derived organoids, which are more useful for modeling tissue development, ASCs-derived epithelial organoids are more inclined to adult tissue repair and regeneration. Consequently, it can be concluded that ASC-derived organoids are built only from tissue compartments with self-renewal and regenerative capacity. 

The development of ASCs-derived organoid technology first originated from the intestine [85,86], and the identification of (Leu-rich repeat-containing G protein-coupled receptor 5) Lgr5 as a marker of Wnt-driven adult gut stem cells was a milestone [87]. Taking the most commonly studied model, intestine organoids, as an example, in the 12 included papers of this systematic review, only 1 study used PSCs [45], while the other 11 studies used ASCs. Among these, six studies [41,42,44,46,48,50] used intestine stem cells, and three studies used pre-isolated stem cells or small intestine crypts, which exhibit revival stem cell markers [13,15,49], while one study used human tissue-derived stem/progenitor epithelial cells via endometrial tissue collection [14]. In most cases, a sequence of growth factor cocktail is required to regulate the differentiation of stem cells in the organoids; therefore, a variety of separate expansion and differentiation media were applied during the culture procedure to induce specific tissue lineages. Curvello et al. reported that a small intestine organoid derived from mouse small intestine crypts, which exhibit revival stem cell markers, offered better conditions for cell survival and proliferation upon exposure to laminin-1 and supplementation with insulin-like growth factor (IGF-1) [13]. The Wnt agonist R-spondin and epidermal growth factor (EGF) are the representative essential growth factors for the in vivo culture of intestinal stem cells. Barker et al. integrated adult Lgr5-positive stem cells into self-organizing, ever-expanding crypt-villus-like intestine organoids by offering abundant growth factors for the intestinal stem cell niche [87]. Collectively, one of the advantages of ASCs-derived organoids is that the time for the procedure is shorter than organoids derived from PSCs. Another advantage is that ASCs are tissue-committed and are more predisposed to differentiate into the designated cell lineage. For the culture of specific ASCs-derived organoids, the culture conditions should be modified, such as the supplementation of appropriate growth factors or small molecules that simulate the molecular microenvironment of specific tissues [81,88].

### 4.2. Current Stem-Cell-Derived Organoid Tissues Using Hydrogels

**Figure 6:**
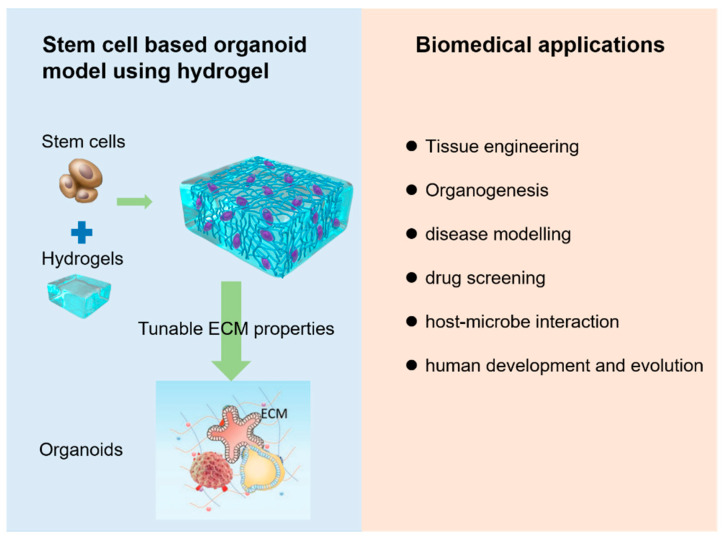
Schematic diagram of constructing stem-cell-derived organoids using hydrogel and their basic biomedical applications.

Strategies for building stem-cell-based organoids using various hydrogels basically depend on the interaction between stem cells and the extracellular microenvironment, which constitutes a dynamic niche that directs the self-renewal, differentiation, and assembly of stem cells in organoids [84]. Figure 6 illustrates the basic process of constructing stem-cell-derived organoids using hydrogels and their basic biomedical applications. The advanced engineering technology could realize the precise control of the geometry input and output flow conditions, nutrient supply, and spatiotemporal and physiochemical stimulation, which would regulate stem cell behavior. In this section, we will describe the main hydrogel-based strategies for constructing stem-cell-derived organoids for different tissue types. 

#### 4.2.1. Intestinal Organoids

Intestinal organoids, also called mini-gut, are currently one of the most studied epithelial organoids and are mostly derived from PSCs and intestinal stem cells (ISCs)-expressing the Lgr5 marker [89]. Recently, researchers investigated functionalized PEG-based hydrogels to simulate the ECM microenvironment and support the organization of organoids [44,47,48]. For example, modified 20 KD 8-arm PEG-macromers with α2β1 integrin-binding peptide (GFOGER) were used to generate intestinal organoids. This synthetic hydrogel system supports human tissue-derived stem/progenitor epithelial cells (ASCs) to form intestinal organoids and is functionally responsive to basolateral stimulation [14]. After modification with arginine–glycine-aspartate (RGD) peptides, a four-arm photosensitive poly (ethylene glycol) (PEG) macromer with maleimide groups (PEG-4MAL)-based hydrogel combined with PSCs, formed an intestinal organoid, which retains the capacity for expansion and differentiation. This organoid had the potential to treat intestinal mucosal wounds in mice [43]. An elegant study developed a round ISCs embedded into RGD–laminin-1–containing PEG-based hydrogel system, which revealed that the underlying mechanisms of epithelial patterning were via yes-associated protein 1 (YAP1) mechanosensing/transduction and Notch signaling [42]. Dynamic gels were reported to precisely control and support the morphogenesis of mouse intestinal stem-cell-derived organoids. A hybrid, stress-relaxing hydrogels made of 8-PEG-Cytosine50 and eight-armed thiol-functionalized PEG (8-PEG-TH) have been shown to be able to adapt to the mechanical force of the microenvironment in the morphogenesis of intestinal organoids. The possible underlying mechanism may involve the stress-relaxing property of these hybrid gels enhancing symmetry breaking and Paneth cell formation relying on the YAP1 signalling pathway, thereby promoting crypt budding [46]. A tunable hydrogel made of plant-based nanocellulose has been developed to support small intestinal organoid growth and budding, which could enable the loading of laminin-1 and IGF-1 to modify the culture conditions [13]. Sachs et al. developed a RGD-functionalized PEG gel (PEG RGD) with essential components for facilitating ISCs differentiation and organoid formation, including laminin-111, collagen IV, hyaluronic acid, and perlecan. This work suggested that matrix stiffness could regulate ISCs expansion and differentiation in colony formation via a YAP1-dependent mechanism [47]. Similarly, Hunt’s team reported a hyaluronan elastin-like protein (HELP) could support the formation of ISCs-based intestine organoids through a YAP1-dependent mechanism [41] responding to the matrix stiffness. Furthermore, the ECM matrix, as well as cellular forces, can decide the size of the stem cell compartment. For intestine organoids, cortical actomyosin contraction can change the surface tensions of cells, thus determining crypt shape [15]. Besides PEG and its derivatives, other hydrogels like Matrigel [48], PAAm [15], alginate [45], collagen-based hydrogel [49], and allyl sulfide photodegradable hydrogel [50] are also commonly utilized hydrogel systems for developing intestine organoids.

#### 4.2.2. Brain and Neural Tube Organoids

The current strategies for developing brain organoids are based on unguided and guided methods. For unguided methodology, this depends on spontaneous self-organization and intrinsic expansion and differentiation potential of PSCs. The guided method needs extra growth factors to guide PSCs to specific committed lineages [90]. In the included paper, Matrigel has been mostly used as a scaffold to support neuroepithelial bud expansion and organoid formation [16,18,53,58]. For example, an elegant study has established PSCs-derived cerebral organoids and mapped human-specific expression in great ape forebrain development using single-nucleus RNA sequencing analysis, which illustrated dynamic divergence in the cortex and chromatin accessibility in the light of gene-regulatory network and genetic change [53]. Similarly, another study generated PSCs-derived cerebral organoids from a chimpanzee and reported that there were 261 genes that were differentially expressed in humans, compared to both chimpanzee organoids and macaque cortex, including “star regulators” PI3K-AKT-mTOR signaling. Moreover, the study revealed that the enhanced activation of this pathway in human radial glia was via two receptors: INSR and ITGB8, which were upregulated specifically in humans [18]. Therefore, stem-cell-derived brain organoids are widely used as a model to establish a platform for systematic analysis of essential molecular networks contributing to human brain development and evolution. The physical properties of the microenvironment have been proven to be an important regulator in organoid construction [45,47]. One study generated a tunable stiffness hydrogel, which modified Matrigel with an interpenetrating network (IPN) of alginate; and it was demonstrated that in this system, stiffer matrixes enabled cell populations to differentiate toward mature neuronal phenotypes, with fewer and smaller neural rosettes [58]. Fattah et al. constructed a single hPSCs-based neural tube organoid model using PEG and found that active mechanical forces regulated by matrix stiffness and cytoskeleton contractility promote organoid growth and orchestrated patterning [60]. Li et al. fabricated a 3D neurospheroid model using a neural stem cell-laden bioink (GelMA matrix), which mimicked the relatively low stiffness of natural non-neuronal brain tissue, thus supporting the formation of neurospheroids and extension of astrocytes [23]. Adding active mechanical forces is a newly-developed strategy for promoting the generation of organoids, as well as adjusting the shape and pattern of organoids, which would benefit their applications in regenerative medicine. A microfluidic device has been applied to promote the survival rate and reduce the variability of organoids. Cho et al. engineered human PSC-derived cerebral organoids utilizing a decellularized human brain tissue-derived brain extracellular matrix (BEM) and dynamic microfluidic systems, and it was shown that this system promoted neurogenesis and resulted in a change of brain organoids at the genomic and functional level [54].

Natural hydrogels can serve as scaffolds for brain organoid formation by regulating the extrinsic microenvironmental cues, including stem-cell–matrix interactions or ECM compositions [91]. For example, Lindborg et al. reported a rapid and robust method for generating hiPSC-derived brain organoids using a defined natural hydrogel made of Sodium hyaluronan (HA-Na) and chitosan, called Cell-Mate3D hydrogel [56]. This hydrogel system exhibited high stiffness, which enhanced the formation of neural tube-like structures, and displayed native brain tissue regions such as the forebrain, midbrain, and hindbrain. In addition, this brain-like organoid could be further applied to adrenoleukodystrophy (ALD) or other neurodevelopmental disease modelling using patient-derived iPSCs [56]. As an essential component of the brain extracellular matrix (ECM), hyaluronic acid (HA) interacts with embedded stem cells via ligand-binding receptors. Li et al. evaluated the physiological function of hiPSCs-based neural cortical spheroids using either HA or heparin plus HA (Hep- HA) hydrogels with different stiffness. The results indicated that stiffer Hep-HA hydrogels showed more caudalizing effect than other groups, with the underlying mechanisms possibly involving Wnt and Hippo/YAP signaling [59]. However, the specific molecular regulatory effect of biomimetic ECM factors for brain or neural tube/neuron organoid requires further investigation.

#### 4.2.3. Retina Organoids

Derived from the neuroectodermal layer, the retina is the vital neural region of the eye that is light-sensitive. Human retinal organoids have been established to investigate retinal development and disease [92,93]. However, current techniques are unable to achieve a five-layered structure similar to a real retina and are unable to carry out a timely transmission of light responses synaptically to the inner retinal layers. Cowan et al. Generated iPSCs-based light-sensitive human retinal organoids and embedded the embryoid bodies into Matrigel. The retinal organoids were proven to have multiple nuclear and synaptic layers, as well as functional synapses [19]. Another study established retinal organoids from CRISPR/Cas9-derived Retinoblastoma 1 (RB1) -null human embryonic stem cells (hESCs). This study has revealed that loss of RB1 drove S-phase entry and led to cellular apoptosis. Meanwhile, RB1 deletion reduced the number of photoreceptors, ganglia, and bipolar cells [20]. 

#### 4.2.4. Cardiac-Tissue Organoids

As an in vitro model for disease modelling, drug screening, and tissue engineering, engineered cardiac organoids derived from human-induced pluripotent stem-cell-derived cardiomyocytes (hiPSC-CMs) show great potential. However, there are some limitations, including structural and functional immaturity of the organoids, which must be overcome [94,95]. To further develop the next generation of patient-specific in vitro cardio-mimetic model systems, Li et al. induced hPSCs to differentiate into ventricular cardiomyocytes (hvCM) and implanted them into collagen-based extracellular matrix hydrogel, thus forming the ventricle-like cardiac organoid chamber (hvCOC) [21]. With organized sarcomeres and myofibrillar microstructures, this cardiac organoid could be electro-mechanically coupled, as well as have fluid-ejecting capacity. RNA sequencing results demonstrated that in this hvCOC system, various vital Ca^2+^-handling ion channels and some important cardiac-specific proteins were upregulated compared with randomly-oriented 2D hPSCs-Ventricular (v) cardiomyocytes (CMs) clusters [21]. The shortcoming of the current heart tissue (EHT) organoid engineering strategy is the inefficient capacity for simulating postnatal myocardial growth, which supports cardiac tissue expansion and increased mechanical load. To overcome this limitation, Lu et al. generated EHTs by assembling hiPSC-CM-derived cardiomyocytes in a low collagen hydrogel with a high density of hiPSCs [22]. Under stretch conditions, hiPSC-CMs exhibited advancement in structural development, including cellular volume, linear alignment, and sarcomere length. In stretched EHTs, RNA-seq analysis suggested that numerous adult-specific cardiomyocyte genes were upregulated. For example, the expression of the alpha-myosin heavy chain (MHC) was downregulated while beta-MHC was upregulated. During growth from the fetal to the adult stage, beta-MHC is predominantly expressed in the ventricle while alpha-MHC expression is reduced. This study showed that medium and high rates of stretch promoted the ratio of beta-MHC by alpha-MHC mRNA, as compared to EHTs of constant length in EHTs [22].

#### 4.2.5. Kidney Organoids

As a significant epithelial organ within the human body, kidney organoids have great potential for investigating human kidney development and disease, especially that affecting glomeruli and renal tubules, as well as for regenerative medicine treatment [96]. Analysis of protein changes within kidney organoids cultured for prolonged durations would enable a better understanding of the development of kidney organoids. A study performed proteomic analysis for extended-cultured kidney organoids and found that the expression of specific ECM components, including types 1a1, 2, and 6a1 collagen and fibronectin (FN) were upregulated [26]. These fibrotic proteins are often found in interstitial regions of aged kidney organoids, which signify kidney pathologies associated with abnormal local accumulation. The researchers then synthesized thiol–ene cross-linked alginate to modulate the ECM of ihPSCs-derived kidney organoids and showed that kidney organoid encapsulation of this synthesized hydrogel decreased deposition of type 1a1 collagen (COL1A1) and other collagen subtypes [26]. Mimicking similar biochemical and biophysical properties (for instance, matrix stiffness) of specific native tissues may provide a strategy for guiding cellular responses and differentiation. Functionalized PAAm hydrogels with tuneable stiffness (ranging from 1 kPa~60 kPa) were fabricated as a scaffold for hPSCs differentiation [24]. RNA sequencing analysis of hPSCs suggested that soft hydrogels upregulated embryo and mesodermal differentiation-related genes, supporting the conclusion that soft ECM may be beneficial for the early stages of embryonic development [24]. The limitation of the current strategy for developing kidney organoids is ineffective vascularization. Kim et al. generated hPSC-derived kidney organoids using a kidney decellularized extracellular matrix (dECM) hydrogel, which enabled extensive vascular network formation and endothelial cell expansion. This study could expand the clinical application of kidney dECM hydrogels in regenerative medicine [25].

#### 4.2.6. Lung Organoids

The COVID-19 pandemic had a significant negative impact on society because of the high infectivity of the severe acute respiratory syndrome coronavirus 2 (SARS-CoV-2). This results in pulmonary symptoms, including mild upper-airway disease and life-threatening acute respiratory distress syndrome (ARDS) [97]. The COVID-19 pandemic has challenged our existing lung model research systems. Recently, the development of human stem-cell-derived lung organoids has provided a powerful tool for the simulation and treatment of lung diseases such as COVID-19 [98]. Researchers are dedicated to exploring the mechanical and chemical properties of various biomaterials, such as the degradation rate, polymer type, and pore size [99]. It is these features that largely ensure transplant efficacy. Dye et al. compared hPSCs-derived lung organoids using poly (ethylene glycol) (PEG) hydrogel, poly(lactide-co-glycolide) (PLG), or polycaprolactone (PCL) scaffolds and found that PEG hydrogel inhibited growth and maturation of lung organoids, with the transplanted lung organoids showing more immature lung progenitors. In contrast, lung organoids embedded on PLG scaffolds or PCL presented more tube-like structures, which was similar to native lung structure and recapitulated the cellular diversity of the adult airway. The mechanism may involve the porous characteristics of microporous scaffold mimicking the airway size and promoting lung airway formation [27]. A better understanding of the vital developmental signaling pathways of hPSCs promotes the generation of ventral-anterior foregut spheroids, which are progenitors of lung organoids. Dye et al. also orchestrated various signaling pathways correlated to embryo development that tightly control organ formation. Firstly, hESCs were treated with Activin-A for endoderm differentiation, followed by Noggin (NOG) and SB431542 (a small molecule TGFβ inhibitor). After these, the cells were exposed to FGF4, and WNT3A (which could inhibit GSK3β), which facilitated 3D spheroid formation. Then the spheroids were transplanted into Matrigel to support organoid growth [28].

#### 4.2.7. Oral Organoids

In this section, various techniques for constructing tooth germ organoids, salivary glands, lingual epithelium organoids, and taste bud organoids are critically examined.

##### Tooth Bud Germ

Tooth replacement regenerative medicine has recently been recognized as possibly feasible in the future, based on spatial-temporal interaction between dental reciprocal epithelium and mesenchyme mesenchymal interactions [100]. Nakao et al. extracted single cells from the tooth-germ-derived dental epithelium (DE) and dental mesenchyme (DM) of the incisor tooth germ at the cap stage in the lower jaws of ED14.5, and reconstituted these cells within a collagen gel droplet, thus generating a bioengineered tooth germ. The structure of this tooth germ organoid resembled a native tooth and exhibited formation of blood vessels and nerve fibres when transplanted under a tooth cavity in vivo. This is the first study to successfully reconstruct an entire tooth germ organoid [101]. 

Two years later, Ikeda et al. made an advancement in the development of tooth germ organoids which could respond to extrinsic mechanical stimulations and pain in cooperation with other maxillofacial tissues when transplanted in vivo [100]. Due to the complexity of tooth structure, the realization of whole-tooth organoid reconstitution is difficult. For an engineered tooth, the stiffness of the hard tissue should resemble the native tooth; the soft tissue should have rich blood circulation and nerve regeneration. To achieve this goal, Smith et al. developed a novel biomimetic tooth bud organoid using post-natal porcine DE, porcine DM progenitor cells, and human umbilical vein endothelial cells (HUVEC) embedded in GelMA hydrogels. They found that this co-cultured tooth germ organoid improved dental cell differentiation and exhibited functional vascularization [29].

##### Salivary Glands

Salivary glands play a pivotal role in oral health; their main functions are saliva secretion, digestion of starch, and swallowing. The difficulties of reconstructing salivary glands include the need to replicate several lineages and simulate duct structure and secretory function [81]. During organogenesis, morphogen signaling, and precise expression of transcriptional factors recapitulate organoid development. A study reported a PSCs-derived salivary glands that incorporated Sox9 and Foxc1 within an organoid culture system. This orthotopically engrafted salivary gland organoid was fully functional in saliva secretion via reconstitution of the central nervous system, which holds great potential in future organ regenerative applications [64]. Shin et al. demonstrated a novel bioengineering technique for promoting the effective organization of human single clonal salivary gland stem cells (SGSCs) via niche-independent 3D microwell culture. Specifically, single clonal SGSCs were induced to aggregate to form 3D spheroids within microwells, which were made of photopatterned PEG hydrogel encapsulating an electrospun polycaprolactone nanofibrous scaffold [65]. Another study isolated human submandibular gland stem/progenitor cells (hSMGepiS/PCs) and induced these cells to form organoids via fibroblast growth factor 10 (FGF10). The induced spheroids mixed with mouse embryonic salivary gland mesenchyme exhibited a mature salivary gland structure and could secrete saliva [31]. 

##### Lingual Epithelial Organoids

The development of lingual epithelial organoids provides new hope for regeneration of the tongue, which could be severely impaired by malignant tongue cancer. Luo et al. have identified keratin 5 (K5) and keratin 14 (K14), which are intermediate filament proteins, as markers for lingual epithelium stem cells (LESCs) and/or progenitors [102]. B cell-specific Moloney murine leukemia virus integration site 1 (Bmi1) was validated to be another novel stem cell marker for LESCs [103]. Bmi1-positive LESCs were cultured with EGF, noggin, and R-spondin1, and suspended in Matrigel. After that, three different types of lingual organoids were found: (1) round-shaped organoids with concentric cell arrangements (round-Org-with-SCs), (2) rugged-shaped organoids with a reticulated cell arrangement (rugged-Org-with-CLs), (3) round-shaped organoids with a reticulated cell arrangement (round-Org-w/o-CLs). In the three lingual organoids, K5 and K14 LESCs/progenitor cells were positively stained, and none of these were stained with positive markers of salivary gland cells or taste bud cells. Among these, only the round-Org-with-SCs presented the representative papillae characteristics, which were marked by a multilayer keratinized epithelium and stratum corneum [30]. However, the limitation of this method for organoid strategy is the heterogeneity of organoids under the same culture condition. Generally, compared with other oral organoids, studies focused on the lingual epithelium organoids are limited and need further exploration. 

##### Taste Bud Organoids

The sense of taste is important to evaluate food quality. Taste buds are independent organs on the tongue. Using the Lgr5/6-EGFP-ires-tamoxifen-inducible Cre recombinase (CreERT2) mouse line, a study has assessed the capacity of leucine-rich repeat-containing G protein-coupled receptor 5 (Lgr5) or Lgr6 to generate taste buds. This organoid model investigated the development of taste bud cells in taste papillae, as well as explored the self-renewal capacity of adult taste stem cells and mature taste cells [32]. Similarly, Aihara et al. established taste bud organoids derived from Lgr5^+^ or CD44^+^ stem/progenitor cells, which exhibited a multi-layered epithelium with stem/progenitor cells and differentiated epithelial taste cells [66]. In the real in vivo environment, especially under acute and chronic inflammation conditions, various cytokines could be significantly induced in subsets of taste bud cells. A study compared the expression of TNF and IL-6, which are the two main inflammatory cytokines, in mouse native taste tissues and in mouse circumvallate stem-cell-derived organoids. The data suggested that this organoid expressed either PLC-β2 or CA4, like the native taste tissue. Moreover, the taste bud organoid expressed Toll-like receptors (TLRs) 2, 3, 4, and 5, all of which are responsible for the recognition of various pathogen molecules [67]. However, since these studies focused on the morphogenesis, development, and function of taste bud organoids, more underlying mechanisms should be further revealed.

#### 4.2.8. Liver Organoids

For severe hepatic failure, orthotopic liver transplantation has demonstrated remarkable regenerative potential to save patients’ lives. However, hepatic sources are extremely limited and difficult to obtain, not to mention expensive surgery, long-term use of immunosuppressive drugs after surgery, and uncertain prognosis [104]. The recent development of liver organoids represents a promising technique to not only enable a deeper understanding of the development and disease pathology of this complicated organ but also holds much potential for regenerative medicine [104]. Chronic inflammation and cancer lead to pathological changes in the liver, as well as changes in the stiffness of the liver tissue. Manipulating the mechanical properties of the hydrogel microenvironment to mimic the physiological stiffness of the native liver is an effective way to construct organoids. Sorrentino et al. modeled the tunable stiffness of PEG (≈1.3 kPa) hydrogels to simulate the stiffness of the fibrotic liver and revealed that the disrupted mechanics of the fibrotic liver restricted the proliferation of liver progenitor cells. The PEG hydrogels were functionalized with laminin-111 and minimal integrin recognition peptide RGDSPG (Arg-Gly-Asp-Ser-Pro-Gly), termed PEG-RGD. Liver organoids were then generated using porcine liver progenitor cells and PEG-RGD, which was highly sensitive to matrix stiffness. The underlying mechanism was not dependent on actomyosin contractility but required activation of YAP and the Src family of kinases (SFKs) [33]. Wauthier et al. generated a porcine liver-derived mesenchymal stem cells-derived liver organoid using soft (~100 Pa) hyaluronan (HA) hydrogels, stiffer hyaluronan hydrogels (coating with silk, ~700 Pa), and a surface layer of HA (~200–300 Pa) that minimizes adhesions from organs and tissues around them. Organoids were transferred into the soft HA hydrogel layer that was on top of the stiffer HA hydrogel, thus constituting multiplayer grafts. The in vivo data revealed that engrafted organoids strongly correlated with matrix-metalloproteinases (MMPs), especially MMP2 (secreted isoforms). This novel patch strategy for liver organoids provides great hope for stem-cell-derived organoid construction for solid organs [68]. A study developed an innovative strategy for one-step synthesis of composite hydrogel capsules (CHCs, composed of a fibrin hydrogel core and an alginate-chitosan composite shell), which boosted hiPSCs-derived liver organoids using an oil-free droplet microfluidic system. This method enables hepatocyte- and cholangiocyte-like cells embedded in liver organoids to grow well. Furthermore, the organoid displayed the predominant features of the human liver, such as urea synthesis and albumin secretion [69]. Although the patch and sandwich structure of liver organoids has advantages, it does not exactly replicate the original architecture. Kim et al. generated liver organoids using alginate hydrogels with primary hepatocytes and MSCs via the 3D printing technique. This technique promoted the expansion capacity of cells while forming multicellular aggregates. Meanwhile, MSCs secreted a variety of paracrine molecules to maintain the morphology of hepatocytes during long-term culture [70].

#### 4.2.9. Pancreas and Islet Organoids

Stem-cell-derived pancreas organoids have been applied to study pancreas development and treat pancreatic damage [105]. Augsornworawat et al. constructed islet organoids using hESC-derived β (SC-β) cells with endothelial cells. Upon plating on Matrigel hydrogel slabs, this novel platform promoted the interaction between SC-β cells and ECs, as well as promoted aggregation of SC-β cells. The islet organoid could secrete insulin under glucose stimulation and express a series of endocrine markers, as well as express a panel of β-cell-associated gene markers [71]. A study investigated the possibility of an innovative droplet microfluidic system for one-step fabrication of hybrid hydrogel capsules, which can be utilized for hiPSC-derived islet organoid engineering. In this system, an array of droplets is applied as templates for the one-step fabrication of binary capsules relying on interfacial complexation of oppositely charged Na-alginate (NaA) and chitosan (CS). This microfluidic system offers much potential for sustainable large-scale production of hiPSCs-derived human islet organoids [72]. Similarly, another study has reported an all-in-water microfluidic system that enabled the one-step fabrication of aqueous-droplet-filled hydrogel fibers (ADHFs). In this system, microfibres could encapsulate pancreatic endocrine progenitor cells induced from hiPSCs, thus forming islet organoids [74]. Candiello et al. demonstrated that the Amikagel hydrogel, which was produced by polymerizing amikacin (an aminoglycoside antibiotic) with PEG-diglycidylether (PEGDE), promoted spontaneous aggregation of hESCs-derived pancreatic progenitor cells (hESC-PP) into robust homogeneous spheroids. Meanwhile, these multicellular islet ‘organoids’ could support the proliferation of HUVECs, laying a solid foundation for the next generation of organoids with rich vascular networks [73].

#### 4.2.10. Muscle and Cartilage Organoids

Skeletal muscle is a highly hierarchical tissue that is crucial for the human body. Recently, newly-developed 3D platforms exhibit much potential for skeletal muscle tissue generation [106]. An interlaced vascular network is crucial for the passive diffusion of nutrients and oxygen in muscle organoids. For example, Gholobova et al. generated muscle organoids through the co-culture of human muscle progenitor cells with HUVECs in a fibrin hydrogel [34]. The artificial skeletal muscle tissues derived from iPSCs (extracted from patients with congenital muscular dystrophies) were implanted in fibrin hydrogels. Using this 3D platform, the severe muscular deformity was simulated, which laid a solid foundation for disease modelling [75]. Regenerative medicine for articular cartilage is currently attractive. Polylactic acid (PLA) and adipic acid dihydrazide (ADH) were used to synthesize hydrogel, which could support adipose-derived stem cell (AdSCs) growth. After long-term culture, this composite could form cartilage organoids [35]. A gelatin-based microcryogels was designed to load MSCs, which could form microniches for 3D printing. Extracellular matrix accumulation in the microniches generated self-assembled composites and can thus be used for cartilage repair [76]. However, more work is still needed to further improve the design of stem-cell-derived muscle and cartilage organoids through the use of hydrogels.

#### 4.2.11. Vascular Network Construction in Organoids

Recently, the construction of a vascular system in the process of organogenesis in vitro is necessary to transport nutrients and remove metabolic wastes, thereby improving tissue survival and thickness. Due to a better understanding of vascular tissue structure and microenvironment, tremendous progress has been made in constructing highly complex biomimetic vascularized tissues and organs using hydrogel materials [11]. The current typical construction strategy is based on an appropriate scaffold embedded with endothelial cells (ECs) prior to in vivo implantation [107]. While pre-grafted ECs have the capacity to form an immature vascular network within the scaffold, the insufficient integration of this network with the host vascular system results in vascular regression within a few days [108].

A methylcellulose-based hydrogel system was used to generate vascular organoids embedded with endothelial cells (ECs)/hPSCs- smooth muscle cells (SMCs). Then, the vascularization function was tested through transplantation in hydrogels made of collagen/fibrinogen/fibronectin [77]. Another study also reported a novel method of using hydrogels as sacrificial scaffolds for cells to self-organize, followed by gentle release. Specifically, a dynamic change in the cross-linking state of alginate could release cell-based structures, and the basic organoid function and stem cell function were not significantly changed. The sacrificial material is deposited, and the sacrificial structure is created by cross-linking the alginate in its patterned state. Cells were then deposited on top to achieve cellular self-organization, and the sacrificial layer was removed. This simple method could allow the formation of highly reproducible multicellular structures on a large scale. Through this strategy, ECs and mesenchymal stem cells (MSCs) could self-organize to form blood-vessel units. When injected into mice, these basic vessel units could rapidly form perfusing vasculature [78]. Acoustic surface standing waves (e.g., the Faraday waves) could drive organoids to form desired patterns and relevant tissue regeneration on-demand via tuned parameters. This technique, called sound-induced morphogenesis (SIM), could control the orderly patterning of ECs and MSCs within a GelMA and fibrin hydrogel to form vascular structures [107]. Therefore, with the rapid development of engineering technology, milder and less invasive strategies for the construction of multiscale vascular networks will be developed.

## 5. Current Challenges and Perspectives

Recent decades have witnessed the dramatic advancement of organoid culture and their applications in modelling disease and development, as well as regenerative medicine, but there are still various challenges that need to be overcome in the future. First of all, organoids are not yet like natural organs. There is still a long way to go before the organoid can replace a real organ and be transplanted into the human body. For different tissues, especially those comprising different germ layers, the construction strategies are complex and diverse, including the utilization of different progenitor cells/stem cells and extracellular matrix scaffolds [81]. Therefore, there are as yet no unified and standardized regulations or protocols for different types of organoids. Even for the same type of organ, there are no uniform production standards, which may raise ethical issues for future clinical applications. At present, research focusing on organoids only concentrates on the aspects of fabrication and its basic functions, but the analyses of molecular and internal mechanisms are not deep enough. It is thus necessary to obtain a more comprehensive and thorough understanding of the underlying molecular mechanisms of tissue development and organogenesis, to drive the novel generation of more mature disease models and tissue/organs.

Secondly, how to obtain a more convenient and cheaper source of stem cells still needs to be addressed. Before embedment with hydrogels or other scaffold materials, it takes a long and tedious experimental process for cells derived from embryonic tissues or pluripotent stem cells to become target cells for organoid culture, not to mention the ethical issues that may be faced during the process [109]. Although scientists have significantly improved the ability to induce pluripotent stem cells to become appropriate cell lineages for specific organoids culture, the current progress for the mass production of organoids is still far from satisfactory [10].

Thirdly, the extracellular matrix (ECM) offers appropriate chemical and mechanical cues for the growth, differentiation, and formation of organoids. Organoid formation involves a dynamic process of stem cell aggregation, expansion, and differentiation, which requires the guidance and interaction of different ECM properties during various stages of tissue engineering. Recent studies have focused on the mechanical properties of hydrogels, especially the stiffness [33,60,63]. However, to realize dynamic regulation of organoid formation, other important physiochemical properties, such as viscoelasticity, topography, and chemical induction by small molecules, should be fully considered [110]. In the future, more attention should be focused on the investigation of advanced biomimetic hydrogels adapted to organoid development at different stages.

In the recent three years, the COVID-19 pandemic can not only cause damage to multiple important organs within the human body but also bring immeasurable economic losses to society. Current efforts to utilize organoids to study SARS-CoV-2 tropism to the whole body, particularly immune cell-mediated host damage, would accelerate drug discovery and vaccine development [12]. In the future, more and more novel techniques and concepts will be used for further development of organoids, including genome editing, immune, vascular organoids, organs-on-a-chip, 3D printing, single-cell multiomics analysis, genome-wide association studies, and deep machine learning.

## 6. Conclusions

In summary, this review outlined the current state of knowledge and research on the various strategies and techniques of culturing organoids, particularly those generated from stem cells and hydrogels. Moreover, this review addresses the potential clinical use of organoids in tissue engineering and disease modelling. Organoids could recapitulate the genetic signature of original tissues, which will enable a better understanding of the developmental processes of vital human organs. With the rapid and further development of various technology platforms, such as gene editing, organ chips, bioprinting, and smart biomaterials, we envision that the development of organoids will enter a new era.

## Figures and Tables

**Table 1:** Summary of stem cell types, hydrogel types, and mechanisms used for engineering different types of organoids.

Tissue	Stem Cell Type	Hydrogel	Hydrogel Type	Mechanism	Ref.
Intestine	mISCs	HELP	Natural hydrogel	High matrix stiffness significantly enhanced ISC expansion through YAP1-dependent mechanism	[41]
	Human tissue-derived stem/progenitor epithelial cells	20 kDa 8-arm PEG macromer	synthetic hydrogel	NA	[14]
	Mouse stem cells (labelled with Lgr5 and Olfm4)	Soft (5 kPa) PAAm	Synthetic hydrogel	The stem cell compartment pushes the ECM and folds through apical constriction, whereas the transit-amplifying zone pulls the ECM and elongates through basal constriction	[15]
	mISCs	RGD–laminin-1–PEG	Synthetic hydrogel	YAP mechanosensing/transduction and Notch signaling	[42]
	hPSCs	Four-armed, maleimide-terminated PEG macromer	Synthetic hydrogel	NA	[43]
	ISCs	RGD-lamin-111-PEG	synthetic hydrogel	NA	[44]
	Mouse small intestine crypts which exhibit revival stem cells markers	Nanocellulose hydrogel	Natural hydrogel	Combined with laminin-1 and supplemented with IGF-1	[13]
	hPSCs	Alginate	Natural hydrogel	RNA-seq	[45]
	ISCs	Hybrid PEG hydrogels	Synthetic hydrogel	Increased symmetry breaking and Paneth cell formation dependent on YAP1	[46]
	mISCs and hISCs	RGD-functionalized PEG gels (with laminin-111, collagen IV, HA acid, and perlecan)	Synthetic hydrogel	High matrix stiffness significantly enhanced ISCs expansion through a YAP-dependent mechanism	[47]
	ISCs	3D printing: PEG elastomers; matrices: patterned Matrigel	Hybrid hydrogel (synthetic-natural)	NA	[48]
	ASCs	A cross-linked collagen hydrogel	Natural hydrogel	Activation of the Wnt signaling pathway to support stem cells while Noggin inhibits BMP to block differentiation	[49]
	ISCs	Allyl sulfide photodegradable hydrogel	Synthetic hydrogel	NA	[50]
Retina	iPSCs	Matrigel	Natural hydrogel	NA	[19]
	hPSCs, including hESC and hiPSC	Matrigel	Natural hydrogel	Exposure to BMP4	[51]
	mESCs	Vinyl sulfone-functionalized 4-arm and 8-arm PEG macromers	Synthetic hydrogel	NA	[52]
	hESCs	Matrigel	Natural hydrogel	Depletion of RB using CRISPR/Cas9 system	[20]
Brain or neural tube	hESCs and iPSC	Matrigel	Natural hydrogel	Human-specific expression was mapped in adult prefrontal cortex using single-nucleus RNA sequencing analysis and identification of developmental differences that persist into adulthood	[53]
	hiPSCs	Matrigel	Natural hydrogel	PI3K-AKT-mTOR signaling	[18]
	Human-induced pluripotent stem cells (iPSCs)	Decellularized human BEM-incorporated hydrogel	Natural hydrogel	RNA-SEQ	[54]
	hESCs	B-ECM hydrogel	Natural hydrogel	protein analysis	[55]
	hPSCs	Cell-Mate 3D hydrogels (HA-Na and chitosan)	Hybrid hydrogel (synthetic-natural)	NA	[56]
	NSCs	GelMA	Synthetic hydrogel	mimic the mechanical modulus of soft tissue while supporting the formation of self-organizing neurospheroids within elaborate 3D networks	[23]
	iPSCs	Collagen hydrogel	Natural hydrogel	RNA-SEQ	[17]
	mESCs	PEG microwells	Synthetic hydrogel	WNT signaling	[57]
	iPSCs	Matrigel was modified with an IPN of alginate	Hybrid hydrogel (natural-natural)	Stiffer matrices skewed cell populations towards mature neuronal phenotype	[58]
	hiPSCs	Heparin and HA Acid	Natural hydrogel	Wnt and Hippo/YAP signaling	[59]
	hPSCs	Matrigel	Natural hydrogel	CRISPR/Cas9; HML-2 activation leads to defective forebrain organoid patterning	[16]
	hPSCs	PEG	Synthetic hydrogel	Organoid response to stretch is mediated extracellularly by matrix stiffness and intracellularly by cytoskeleton contractility and planar cell polarity	[60]
	mESCs	PEG	Synthetic hydrogel	NA	[61]
	hESCs	Alginate	Natural hydrogel	NA	[62]
Heart or cardiovascular organoid	hiPSCs	Collagen	Natural hydrogel	NA	[22]
	hPSCs	Collagen-based ECM hydrogel	Natural hydrogel	Upregulation of key Ca^2+^-handling, ion channel, and cardiac-specific proteins	[21]
	ESCs	Collagen-conjugated PAAm hydrogels (natural-synthetic)	Hybrid hydrogel (synthetic-natural)	Modulating the stiffness of a cell adherent hydrogel	[63]
Kidney	hiPSCs	Thiol–ene cross-linked alginate hydrogel	Synthetic hydrogel	A reduction of abnormal type 1a1 collagen expression	[26]
	hPSCs	A kidney dECM hydrogel	Natural hydrogel	CRISPR/Cas9	[25]
	hPSCs	Natural chick CAM	Natural hydrogel	Physiologically relevant soft microenvironment could favor the differentiation of kidney organoids	[24]
Lung	hPSCs	PEG; PCL or PLG	Synthetic hydrogel	Microporous scaffold can affect lung airway formation, airway size, and explant size	[27]
	hPSCs, hESCs and hiPSCs	Matrigel	Natural hydrogel	RNA-SEQ	[28]
Tooth germ organoids	pDM progenitor cells	GelMA	Synthetic hydrogel	NA	[29]
Salivary gland	ESCs	Matrigel	Natural hydrogel	Sox9 and Foxc1	[64]
	Human single clonal SGSCs	PEG with an electrospun PCLnanofibrous scaffold	Synthetic hydrogel	NA	[65]
	hSMGepiS/PCs	Matrigel	Natural hydrogel	FGF10	[31]
Lingual epithelium organoids	Adult epithelial stem cells	Matrigel	Natural hydrogel	EGF, noggin, and R-spondin 1	[30]
Taste bud organoids	Lgr5+ or Lgr6+ taste bud stem cells	Matrigel	Natural hydrogel	R-spondin 1, Noggin, Jagged 1, Y27632, N-acetylcysteine, EGF, N2, and B27	[66]
	Adult taste stem/progenitor cells	Matrigel	Natural hydrogel	Wnt, EGF, R-spondin 1, and Noggin	[32]
	Mouse circumvallate stem cells	Matrigel	Natural hydrogel	Toll-like receptors (TLRs)-mediated inflammatory cytokine expression	[67]
Liver	Liver progenitor cells	PEG-RGD	Synthetic hydrogel	Organoid growth is stiffness-sensitive, independent of actomyosin contractility, and requiring instead activation of the SFKs and YAP	[33]
	Porcine liver-derived MSCs	HA hydrogels	Natural hydrogel	Expression of MMPs	[68]
	hiPSCs	CHCs composed of a fibrin hydrogel core and an alginate-chitosan composite shell	Hybrid hydrogel (natural-natural)	NA	[69]
	MSCs	alginate hydrogels	Natural hydrogel	NA	[70]
Pancreas and islet	hESCs	Matrigel	Natural hydrogel	Physical culture conditions greatly influence the interactions among these cell types	[71]
	hiPSCs	Na-alginate (NaA) and chitosan	Hybrid hydrogel (natural-natural)	NA	[72]
	hPSCs	amikagel	Synthetic hydrogel	Amikagel-induced hESC-PP spheroid formation enhanced pancreatic islet-specific Pdx-1 and NKX6.1 gene and protein expression while also increasing the percentage of committed population	[73]
	hiPSCs	ADHFs	Natural hydrogel	The established system enabled the formation of functional human islet organoids in situ by encapsulating pancreatic endocrine progenitor cells within microfibers	[74]
Muscle	iPSCs	Fibrin hydrogels	Natural hydrogel	NA	[75]
	human muscle progenitor cells are cocultured with HUVECs	Fibrin hydrogel	Natural hydrogel	NA	[34]
Cartilage	AdSCs	PLA, ADH	Hybrid hydrogel (synthetic-synthetic)	TGF-β1 and IGF-1	[35]
	MSCs	Gelatin-based microscopic hydrogel (microcryogels)	Natural hydrogel	Self-assembly was induced by the connection of microniches through ECM accumulation secreted by MSCs	[76]
Vascular organoids	hPSCs	Methylcellulose-based hydrogel system	Natural hydrogel	NA	[77]
	co-culture of ECs and MSCs (of either mouse or human origin)	Alginate microwells	Natural hydrogel	NA	[78]
	HUVEC and hMSCs	GelMA and fibrin gel, with TCP particles	Hybrid hydrogel (synthetic-natural)	NA	[79]

## Abbreviations

ADH: adipic acid dihydrazide; AdSCs: adipose-derived stem cells; ASCs: adult stem cells; AL: alginate; AHDFs: aqueous-droplet-filled alginate calcium hydrogel fibers; BEM: brain extracellular matrix; BMP: bone morphogenetic protein; BMSCs: bone marrow-derived MSCs; CAM: chorioallantoic membrane; CH: chitosan; COL: collagen; dECM: decellularized extracellular matrix; ECM: extracellular matrix; EHT: engineered heart tissue; EL: elastin; ESCs: embryonic stem cells; gelMA: gelatin-methacryloyl; HA: hyaluronic acid; HELP: hyaluronan elastin-like protein; IGF-1: insulin like growth factor 1; hPSCs: human pluripotent stem cells; hSMGepiS/PCs: human submandibular gland stem/progenitor cells; HUVECs: human umbilical vein endothelial cells; iPSCs: induced pluripotent stem cells; IPN: interpenetrating network; MSCs: mesenchymal stromal cells;MMPs: matrix-metalloproteinases; NSCs: neural stem cells; PAAm: polyacrylamide gels; pDM: porcine dental mesenchymal; PEG: polyethylene glycol; PLA: polylactic acid; PLG: poly(lactide-co-glycolide); PCL: polycaprolactone; PSCs: pluripotent stem cells; SFKs: Src family of kinases; SGSCs: salivary gland stem cells; TGF: transforming growth factor; TLRs: Toll-like receptors; YAP1: yes-associated protein 1.
